# Factors affecting the delivery of community pharmacist-led medication reviews: evidence from the MedsCheck annual service in Ontario

**DOI:** 10.1186/s12913-016-1888-2

**Published:** 2016-11-21

**Authors:** Petros Pechlivanoglou, Lusine Abrahamyan, Linda MacKeigan, Giulia P. Consiglio, Lisa Dolovich, Ping Li, Suzanne M. Cadarette, Valeria E. Rac, Jonghyun Shin, Murray Krahn

**Affiliations:** 1 Toronto Health Economics and Technology Assessment Collaborative, University of Toronto, Toronto, ON Canada; 2 The Hospital for Sick Children Research Institute, Peter Gilgan Centre for Research and Learning, 686 Bay st, Toronto, M5G0A4 ON Canada; 3 The Institute for Health Policy Management and Evaluation (IHPME), University of Toronto, Toronto, ON Canada; 4 Leslie Dan Faculty of Pharmacy, University of Toronto, Toronto, ON Canada; 5 Institute for Clinical Evaluative Sciences , Toronto, ON Canada; 6 Department of Family Medicine, McMaster Innovation Park University, Hamilton, ON Canada; 7 University Health Network, Toronto General Hospital, Toronto, ON Canada

**Keywords:** Medication review, Pharmacy, Predictors, Community, Elderly

## Abstract

**Background:**

Medication reviews have become part of pharmacy practice across developed countries. This study aimed to identify factors affecting the likelihood of eligible Ontario seniors receiving a pharmacy-led medication review called MedsCheck annual (MCA).

**Methods:**

We designed a cohort study using a random sample of pharmacy claims for MCA-eligible Ontario seniors using linked administrative data from April 2012 to March 2013. Guided by a conceptual framework, we constructed a generalized-estimating-equations model to estimate the effect of patient, pharmacy and community factors on the likelihood of receiving MCA.

**Results:**

Of the 2,878,958 eligible claim-dates, 65,605 included an MCA. Compared to eligible individuals who did not receive an MCA, recipients were more likely to have a prior MCA (OR = 3.03), receive a new medication on the claim-date (OR = 1.78), be hypertensive (OR = 1.18) or have a recent hospitalization (OR = 1.07). MCA recipients had fewer medications (e.g., OR = 0.44 for ≥12 medications versus 0–4 medications), and were less likely to receive an MCA in a rural (OR = 0.74) or high-volume pharmacy (OR = 0.65).

**Conclusions:**

The most important determinant of receiving an MCA was having had a prior MCA. Overall, MCA recipients were healthier, younger, urban-dwelling, and taking fewer medications than non-recipients. Policies regarding current and future medication review programs may need to evolve to ensure that those at greatest need receive timely and comprehensive medication reviews.

## Background

Inappropriate medication prescribing and use can result in adverse health outcomes for patients, and increased costs to healthcare systems [1–3], especially for individuals receiving multiple medications for multiple conditions. In response, public drug plans in several countries now reimburse community pharmacies for one-on-one reviews of patients’ list of medications [4–7]. Broadly, medication reviews aim to improve outcomes through educating patients about their medications and health conditions, promoting safe medication use, improving adherence, and establishing an effective patient-pharmacist collaboration [7, 8].

There is no standardized approach to medication reviews. Across the programs implemented so far, substantial variation is observed in referral mechanisms, eligibility criteria, pharmacist training, reimbursement schemes, and frequency, content and location of the reviews [5, 9]. In the United Kingdom (UK) medication reviews have been classified into three types: prescription review (a review of patients’ prescription or medication records); compliance or concordance review (explores patients’ medication-taking practices, knowledge and beliefs); and clinical medication review (conducted with access to patients’ medical record and with the patient present) [7].

In 2007, the Ontario Ministry of Health and Long-Term Care (MOHLTC) launched MedsCheck (MC), the first government-funded, community pharmacy-led medication review program in Canada [5]. The stated purpose of the MC service is to help patients better understand their medication therapy and ensure that medications are taken as prescribed (MC Program Guidebook 2008) [10]. Therefore, MC is a compliance or concordance review. Residents of Ontario taking three or more prescription medications for a chronic condition are eligible for a MC. There are no additional eligibility requirements for the patient, pharmacist, or community pharmacy. No formal referral is required; most commonly, community pharmacists approach patients about receiving a MC. The service must be conducted as a one-on-one interview between patient and pharmacist in an acoustically private area of the pharmacy; an appointment is not required. At the time of this study, the results of the MC had to be shared with the patient/caregiver in the form of a comprehensive medication list (prescription and nonprescription drugs plus natural health products) and, when appropriate, with the physician. The first service provided through the MC program was MC Annual (MCA), as described above, and reimbursed with a fee of $50 (increased to CAN $60 in 2010). Over time the scope of MC expanded, with MC Follow up, MC Diabetes, MC at Home and MC Long-term Care (LTC) medication reviews being introduced at different fee levels.

Since its initiation, the utilization of MCA has increased. Until 2013 about 1.5 million Ontario residents received MCA at least once [11]. In fiscal year 2012–13 about 327,000 MCAs were provided at an estimated cost of CAN$22.3 million [11]. However, there has been no formal evaluation of the program to date. This study represents one component of a comprehensive evaluation consisting of: i) a description of the characteristics of patients receiving MCA; ii) factors associated with receiving MCA; and iii) health outcomes associated with MCA. In the study reported herein we attempted to understand the patient, pharmacy and community factors affecting the likelihood of receiving an MCA among eligible Ontario seniors.

## Methods

We conducted a population-based cohort study of linked administrative and clinical data to investigate the likelihood that eligible Ontario seniors would receive an MCA. Because the Ontario Drug Benefit Program (ODB) provides prescription drug coverage primarily for Ontario residents who are over 65 years old, drug claims data were only available for this population. Therefore, the study population was limited to Ontario seniors.

The databases used are hosted at Ontario’s Institute for Clinical Evaluative Sciences (ICES) and include information for all Ontario residents on: i) demographic and vital statistics (through the Registered Persons Database), ii) comorbidities and utilization of inpatient and outpatient healthcare services (Ontario Health Insurance Plan Physician Claims database, Discharge Abstract Database and National Ambulatory Care Reporting System Database of the Canadian Institute of Health Information), iii) prescription medication and other Ontario government-funded community pharmacy services (ODB database). These datasets were linked using unique encoded identifiers and analyzed at the Institute for Clinical Evaluative Sciences (ICES). The full data set creation plan is available from the authors upon request.

We limited the analysis to the period between April 1, 2012 and March 31, 2013 as we expected Ontario community pharmacies to have adapted their practice to MCA by then. Also, this represented a period of relative stability, following a period (2010–2011) in which reimbursement for a number of new pharmacy-led medication therapy management (MTM) services were introduced by the MOHLTC. We excluded claims that were submitted after the date of death of an individual, claims for long-term care residents and for MC services other than MCA. For computational feasibility purposes, we limited our study to a random 20 % sample of the seniors identified in the sample period in the ODB database. The sample selection was based on random draws without replacement from the unique personal identifiers associated with every eligible senior in the ODB database.

We defined a “claim-date” as any date for which seniors had a pharmacy claim. These claims could represent dispensing of prescription medication, or providing another ODB-covered pharmacist service, such as an MCA. We defined individuals in the ODB as eligible for an MCA on a given claim-date if they were at least 66 years old (versus the ODB eligibility date of age 65 to ensure 1 year of look-back), had claims for three or more chronic prescription medications over the past 6 months, and had not received any MC services in the past year. Only eligible ODB claim-dates during the study period were considered. Finally, we assumed that MCAs that occurred within 14 days from a prescription or a pharmacist service claim-date were the result of an appointment arranged at this claim-date.

As the MC program does not indicate which medications are considered chronic, or for a chronic condition, we relied on expert opinion to differentiate between chronic and non-prescription medications. The list of chronic prescription medications covered by the ODB program was defined by two pharmacists who independently reviewed the ODB Formulary and excluded drug identification numbers (DINs) of non-chronic medications. Disagreements were resolved through discussion. Over-the-counter and chronic prescription medications not covered by the ODB program were not considered in our patient selection process or analysis.

### Conceptual framework and predictor variables

**Fig. 1:**
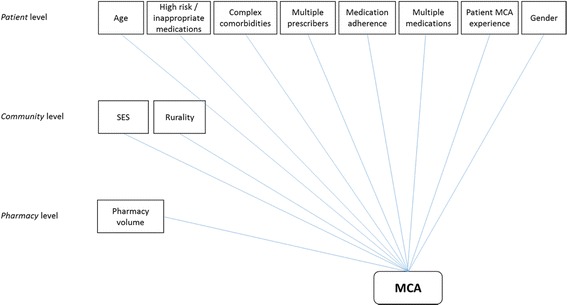
Conceptual framework of observed and latent predictors for utilization of MCA services MCA, MedsCheck Annual; SES, socio-economic status

After reviewing relevant policy documents and the published literature, we developed a conceptual framework that summarized potential factors associated with the likelihood of receiving an MCA (Fig. 1). Factors were classified into patient-level (age, sex, comorbidities, past experience with similar services), pharmacy-level (volume), and community-level (rurality, socio-economic status (SES)). A similar conceptual framework has been used in other healthcare service studies using Ontario population-level databases [12].

The MC Guidebook lists types of patients most likely to benefit from an MCA as those with a history of medication non-compliance, with a significant medication regimen change, prescribed a high-risk medication, or recently discharged from hospital [10]. Research evidence supports these characteristics as risk factors for drug-related morbidity and mortality [13]. Other important factors are polypharmacy and multiple medical conditions [1]. Therefore, we included nonadherence (measured using the proportion of days covered), use of high-risk or potentially inappropriate medications, number of medications, and recent hospitalization, as potential predictors of receiving MCA (see Supplemental Digital Content (SDS) 1, for definitions of these variables). These variables were defined as patient-level factors.

Patient demographic factors such as age have been found to have a positive correlation with receiving MTM services in the US [14] possibly because older adults are more likely to have multiple medications, chronic conditions and multiple treating physicians. Being a woman has been associated with a higher probability of receiving an MTM service [14] which is in accordance with previous findings indicating that fewer men seek health-related advice [15]. The literature is conflicting regarding the effect of the number of medications on the probability of receiving a medication review. In a qualitative study in the UK, pharmacists reported that they were less likely to provide Medicines Use Reviews (MURs) to patients who had more medications, complicated medication regimens or complex conditions because they perceived these consultations as lengthy [16]. In a survey of Medicare Part D beneficiaries in the USA, respondents who received a comprehensive medication review (CMR) as part of their MTM program were prescribed a significantly higher number of medications than those who had not [17]. Other patient-level factors included in our model are comorbidity type; overall level of comorbidity, using the Aggregated Diagnosis Groups (ADGs) metric; number of prescribers; use of high risk and potentially inappropriate medications; and prior experience with MCA. A diagnosis of dementia was used to represent patients who would be less likely to receive an MCA because of inability to visit a pharmacy and/or to participate in a MCA. Comorbidities were defined using International Classification of Diseases 10th Revision (ICD-10) diagnosis codes. The definition of high-risk medication was based on the list of high-alert medications in the community proposed by the Institute for Safe Medication Practices and two recent Canadian reports [18–20] on adverse drug-related emergency department visits and hospitalizations.

Pharmacy prescription volume and ownership type have been identified as pharmacy-level factors influencing provision of medication reviews, with chain pharmacies being more likely to provide a medication review [21]. Although our study could not distinguish between chain and independent pharmacies, we anticipated that pharmacies with higher ODB prescription volumes would be more likely to be chain pharmacies.

Community-level factors included socioeconomic status (SES) and rurality. Lower SES has been associated with lower uptake of medication reviews [21]. As proxies for SES we used the neighborhood income quintile and the material deprivation dimension of the Ontario Marginalization Index [22]. Rurality was measured by the Rurality Index of Ontario (RIO), which incorporates information on community population density and access to referral centres [23]. Rurality and SES were based on patients’ enumeration area using 2006 Canada Census data.

### Statistical methods

Descriptive statistics were applied to both categorical and continuous variables. Differences in frequencies were compared using the *χ* ^2^ test while mean differences were compared using an independent t-test. In order to evaluate the independent effects of patient-, pharmacy- and community-level factors on the likelihood of receiving an MCA, we used a Generalized Estimating Equations (GEE) framework to take into account the repeated nature of the data, since there can be multiple claim-dates for each patient, and multiple patients within each pharmacy [24, 25]. The variable corresponding to receiving an MCA was modeled using a binomial distribution. We set the statistical significance level at 0.05 and corrected for type I error due to multiple testing using the Bonferroni correction [26]. The quasi-likelihood under the independence model criterion (QIC) was used to inform the correlation structure [27]. Model selection was based on the QIC and our conceptual framework. In order to illustrate our findings in a more meaningful way we developed four contrasting scenarios of hypothetical Ontario seniors and estimated the probability that each senior would receive an MCA.

## Results

### Descriptive statistics

**Fig. 2:**
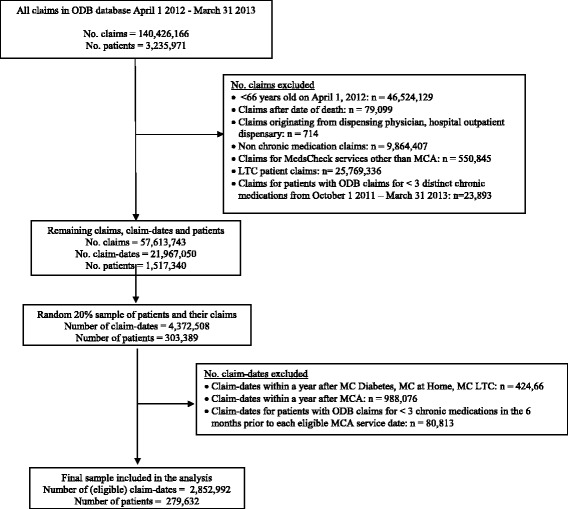
Flowchart of study sample selection. ODB: Ontario Drug Database, MCA: Medscheck Annual, LTC: long-term care

A total of 279,632 seniors in the 20 % random sample were identified as eligible to receive an MCA between April 1, 2012 and March 31, 2013, at least once (Fig. 2). These patients were associated with 2,852,992 eligible claim-dates and 3,884 pharmacies with unique billing numbers (almost all unique Ontario pharmacies in 2012–2013). Approximately 23.5 % (*n* = 65,605) of the eligible seniors received MCA within the study period.

Table 1 presents descriptive statistics for patient-, community- and pharmacy-level characteristics of seniors with MCA claims compared to those with no MCA claim on the eligible claim date. Most MCAs were provided in urban/suburban areas (i.e. RIO < 9) and in communities with a lower deprivation index. More MCA recipients were men and younger seniors receiving fewer medications from fewer prescribers, and with fewer inappropriate or high risk medications. In addition, pharmacies with high prescription claims volume were much less common in the MCA group than in the non-recipient group.

**Table 1:** Patient, pharmacy and community level characteristics on MCA-eligible pharmacy claim-dates

Characteristics	MCA claims *n* = 65,605	Non-MCA claims *n* = 2,787,387	*P*-value
Patient-level characteristics defined at the first claim-date			
Age (years), *n* (%)			
66–70	16,717 (25.5)	674,825 (24.2)	*
71–75	16,444 (25.1)	617,611 (22.2)	
76–80	14,754 (22.5)	572,702 (20.6)	
81–85	10,741 (16.4)	474,862 (17.0)	
86+	6,949 (10.6)	447,387 (16.0)	
Female, *n* (%)	36,471 (55.6)	1,632,655 (58.6)	*
Recent immigrant (<15 years), *n* (%)	2,086 (3.2)	65,645 (2.4)	*
Hypertension, *n* (%)	55,035 (83.9)	2,286,761 (82.0)	*
Ischemic heart disease, *n* (%)	12,111 (18.5)	534,862 (19.2)	*
Heart failure, *n* (%)	7,281 (11.1)	445,334 (16.0)	*
Diabetes, *n* (%)	17,252 (26.3)	1,054,580 (37.8)	*
COPD/asthma, *n* (%)	20,650 (31.5)	980,904 (35.2)	*
Osteoporosis, *n* (%)	12,875 (19.6)	545,372 (19.6)	0.709
Cancer, *n* (%)	11,876 (18.1)	500,281 (17.9)	0.311
Depression, *n* (%)	1,857 (2.8)	110,137 (4.0)	*
Dementia, *n* (%)	2,388 (3.6)	216,747 (7.8)	*
Prior MCA, *n* (%)	37,820 (57.6)	944,962 (33.9)	*
Adherent to ACEi/ARB/statin, *n* (%)			
No	4,388 (6.7)	164,301 (5.9)	*^a^
Yes	45,390 (69.2)	1,907,223 (68.4)	
NA (not receiving)	15,827 (24.1)	715,863 (25.7)	
Number of ADGs in past 2 years, mean (SD)	8.1 (3.7)	8.4 (3.9)	*
Number of ED admissions in the last year, mean (SD)	0.6 (1.4)	0.829 (1.8)	*
Number of hospitalizations in the last year, mean (SD)	0.5 (0.9)	0.542 (1.1)	*
Number of physicians in the last year, mean (SD)	7.9 (6.7)	8.6 (7.8)	*
Patient-level characteristics defined at each claim-date			
New hospitalization/ED admission in past 60 days, *n* (%)	9,921 (15.1)	457,454 (16.4)	*
Use of high risk medication in past 6 months, *n* (%)	52,928 (80.7)	2,314,441 (83.0)	*
Use of PIMs in past 6 months, *n* (%)	27,532 (42.0)	1,406,024 (50.4)	*
New prescription medication at claim-date, *n* (%)	16,026 (24.4)	396,030 (14.2)	*
Number of physicians in past 3 months, mean (SD)	5.2 (5.0)	5.3 (5.4)	*
Number of unique medications in past 4 months, mean (SD)	6.7 (3.5)	7.8 (4.3)	*
Number of unique medications in past 4 months (quintiles), *n* (%)			
0–4	19,232 (29.3)	668635 (24.0)	*
5–6	17,672 (26.9)	553817 (19.9)	
7–8	12,529 (19.1)	507791 (18.2)	
9–11	10,111 (15.4)	544720 (19.5)	
12–42	6,061 (9.2)	512424 (18.4)	
Pharmacy-level characteristic			
Pharmacy volume (quintiles), *n* (%)			
1 (lowest)	4,940 (7.8)	137,937 (5.1)	*
2	9,147 (14.5)	283,112 (10.5)	
3	13,661 (21.6)	461,396 (17.2)	
4	16,757 (26.5)	675,308 (25.1)	
5 (highest)	18,726 (29.6)	1,129,756 (42.0)	
Community-level characteristics			
Rurality index of Ontario, *n* (%)			
0–9 (major urban)	47,470 (72.8)	1,744,607 (63.2)	*
10–44 (non-major urban)	14,210 (21.8)	803,936 (29.1)	
45+ (rural)	3,559 (5.5)	211,667 (7.7)	
Income quintiles, *n* (%)			
1 (lowest)	11,582 (17.7)	569,300 (20.5)	*
2	13,593 (20.8)	583,981 (21.0)	
3	12,999 (19.9)	546,306 (19.7)	
4	13,397 (20.5)	542,397 (19.5)	
5 (highest)	13,825 (21.1)	533,984 (19.2)	
Material deprivation quintiles, *n* (%)			
1 (lowest)	16,114 (24.8)	602,791 (22.0)	*
2	15,327 (23.6)	610,747 (22.3)	
3	13,919 (21.4)	591,068 (21.5)	
4	11,192 (17.2)	515,031 (18.8)	
5 (highest)	8,365 (12.9)	424,022 (15.5)	

*P-value < 0.001. ^a^‘No’ versus ‘Yes’

*ACEi* indicates angiotensin converting enzyme inhibitor; *ADG* Aggregated Diagnosis Group based on the Johns Hopkins Ambulatory Care Group case-mix system, *ARB* angiotensin receptor blocker, *COPD* chronic obstructive pulmonary disease, *ED* emergency department, *MCA* MedsCheck Annual, *NA* not applicable, *PIM* Potentially inappropriate medication, *SD* standard deviation

### Predictors of MCA utilization

The final GEE model is presented in Table 2. The model takes into account the within-patient correlation and assumes a structure of exchangeable correlations across patients. In accordance with our hypotheses, we found that having a prior MCA service (OR = 3.03; 95% confidence interval (CI), 2.98–3.09), having a claim for a new prescription on the eligible claim-date (OR = 1.78; 95% CI, 1.74–1.81), or using a high risk medication (OR = 1.09; 95% CI, 1.07–1.12) increased the likelihood of receiving an MCA. Patients with dementia or depression were less likely to receive an MCA (OR = 0.57; 95% CI, 0.55–0.60 and OR = 0.90; 95% CI, 0.86–0.95 respectively) while patients with hypertension were more likely (OR = 1.18; 95% CI, 1.15–1.21). However, the likelihood of receiving an MCA was lower for older patients, patients with heart failure (HF) (OR = 0.88; 95 % CI, 0.86–0.91), women (OR = 0.90; 95 % CI, 0.89–0.92), those receiving either potentially inappropriate medications (OR = 0.90; 95 % CI, 0.88–0.91) or a large number of medications, those visiting a high-ODB claims volume pharmacy and those living in a rural community (Table 2). Statistically significant differences were observed for several other covariates (e.g. adherence, number of physicians) although their magnitude was not considered clinically meaningful.

**Table 2:** Results of GEE model analysis to identify patient, pharmacy, and community factors associated with receiving an MCA service

Parameter	Odds Ratio	95 % CI		*P*-value
Intercept	0.029	0.028	0.031	*
Age, years (reference: 66–70)				
71–75	0.999	0.976	1.022	
76–80	0.989	0.966	1.014	
81–85	0.922	0.897	0.948	*
86+	0.716	0.694	0.740	*
Female	0.904	0.888	0.919	*
Recent immigrant <15 years	1.016	0.969	1.066	
Hypertension	1.176	1.149	1.205	*
Ischemic heart disease	1.045	1.021	1.070	*
Heart failure	0.884	0.859	0.910	*
Diabetes	0.614	0.601	0.627	*
COPD/asthma	0.978	0.960	0.997	
Depression	0.903	0.858	0.951	*
Dementia	0.574	0.548	0.600	*
Prior MCA	3.032	2.979	3.086	*
Adherence to ACEi/ARB/statin (reference: non–adherent)				
Adherent to ACEi/ARB/statin	1.043	1.008	1.080	
Not receiving ACEi/ARB/statin	0.897	0.865	0.931	*
Number of ADGs in past 2 years	0.994	0.991	0.997	*
Number of ED visits in the last year	0.981	0.972	0.989	*
Number of hospitalizations in the last year	0.990	0.979	1.001	
Number of physicians in the last year	0.994	0.992	0.996	*
New hospitalization/ED admission in past 60 days	1.065	1.041	1.090	*
Use of high risk medication in past 6 months	1.091	1.066	1.116	*
Use of PIMs in past 6 months	0.896	0.880	0.912	*
New prescription medication at claim-date	1.776	1.744	1.808	*
Number of physicians in past 3 months	1.013	1.011	1.015	*
No. unique medications in the past 4 months (reference category: 0–4)				
5–6	1.010	0.988	1.033	
7–8	0.803	0.782	0.824	*
9–11	0.641	0.622	0.660	*
11+	0.443	0.427	0.460	*
Pharmacy volume^a^ (reference category: lowest)				
2	0.884	0.851	0.918	*
3	0.870	0.839	0.901	*
4	0.784	0.757	0.812	*
5 (highest)	0.649	0.626	0.672	*
Rurality index of Ontario (reference category: major urban, 0–9)				
10–44 (non-major urban)	0.754	0.739	0.770	*
45+ (rural)	0.737	0.710	0.765	*
Income quintile (reference category: lowest)				
2	1.056	1.028	1.085	*
3	1.051	1.023	1.080	*
4	1.058	1.029	1.087	*
5 (Highest)	1.079	1.050	1.109	*

*P value < Bonferroni corrected α: 0.0012. ^a^Quantiles formed from the number of claims per pharmacy from April 1^st^ 2011 to March 31^st^ 2012

*ACEi* indicates angiotensin converting enzyme inhibitor, *ADG* Aggregated Diagnosis Group based on the Johns Hopkins Ambulatory Care Group case-mix system, *ARB* angiotensin receptor blocker, *CI* confidence interval, *COPD* chronic obstructive pulmonary disease, *ED* emergency department, *MCA* MedsCheck Annual, *PIM* Potentially inappropriate medication

### Patient scenarios

Table 3 provides plausible profiles for four hypothetical seniors, along with their estimated likelihood of receiving an MCA. Scenario A describes a senior whose characteristics are those of a high risk of MCA senior. The likelihood of receiving an MCA on an eligible date is around 11.5 %.In contrast, Scenario D describes a senior with less favorable profile for MCA. Compared to the patient in Scenario A, she was estimated to be ten times less likely (1 %) to receive an MCA on an eligible claim-date. Scenarios B and C illustrate the likelihood of receiving an MCA for individuals with other plausible covariate combinations.

**Table 3:** Scenarios of hypothetical seniors, and associated probability of receiving an MCA service on an eligible claim-date

	Scenario A	Scenario B	Scenario C	Scenario D
Age (years)	66	80	70	80
New prescription medication at claim-date	Yes	No	Yes	No
Number of chronic medications	3	9	5	9
Number of physicians in the last year	1	3	2	3
Number of comorbidities	2	5	3	8
Hypertension	Yes	Yes	Yes	No
Heart failure	No	Yes	No	Yes
Recent hospitalization (≤60 days)	No	Yes	No	Yes
Use of a high risk medication	No	No	No	Yes
Use of a potentially inappropriate medication	No	Yes	No	Yes
Adherent use of ACEi/ARB/statins	Yes	Yes	Yes	Yes
Prior MCA	Yes	Yes	No	No
Sex	Male	Female	Male	Female
Residence setting	Urban	Urban	Rural	Urban
Income level (quintiles)	Highest	Highest	Lowest	Highest
Pharmacy volume (quintiles)	Highest	Highest	Lowest	Highest
Probability of receiving an MCA service (%)	11.5	4.9	4.7	1.0

*ACEi* indicates Angiotensin-converting-enzyme inhibitor, *ARB* Angiotensin II receptor blocker

## Discussion

This study aimed at understanding factors affecting the likelihood of receiving an MCA among Ontario seniors. Although some groups identified as likely to benefit from a medication review did receive an MCA (e.g. those with a prescription for a new medication), the profile of seniors receiving MCAs was generally that of healthier, younger, urban-living seniors on relatively fewer medications.

### Patient factors

Previous MCA experience was the strongest predictor of receiving an MCA. This finding could be the result of patients’ approaching the pharmacist for another MCA after a positive prior experience. In a US study 24 % of individuals who had received a medication review (brown bag review) responded positively when asked if they would have a review annually [28]. In addition, from a pharmacist’s perspective, experienced MCA users may be easy to identify through the pharmacy’s patient profile system, and engage since they have responded positively to MCA in the past.

Being on multiple medications or being older had a strong negative correlation with the likelihood of receiving an MCA. These findings were contrary to what we expected based on the Program Guidebook’s list of patients most likely to benefit from a MC [10] and evidence from an analysis of a US cohort of Medicare beneficiaries where MTM service enrollees had more prescriptions than eligible non-MTM enrollees. [17] Regarding age, a positive association was observed in a previous study [14] possibly because the initiation of the medication review was purely at the discretion of the patient. It is possible that because MCAs provided to older seniors with multiple medications are likely to be more time-consuming and complex, Ontario pharmacists might show preference in offering the service to younger and healthier individuals, as has been reported with the MUR program in the UK [16]. Another explanation could be that sicker or older patients might not be physically capable of visiting the pharmacy to pick up their own prescriptions and thus could not be approached in person about receiving a MCA.

We postulated that the use of high-risk and potentially inappropriate medications might be one of the triggers for initiating MCA. However, we observed that the use of high-risk medications increased the odds while the use of potentially inappropriate medications decreased the odds of receiving an MCA. The characteristics of users of these two medication groups could differ, with users of potentially inappropriate medications being older and more likely to have mental health issues. In the US MTM program, having a high risk medication (based on 2002 Beers criteria) increased the likelihood of enrollment in a medication review program [29].

In our study the average number of ADGs (comorbidity groups) was statistically significant but not clinically different between MCA and non-MCA groups. Having HF, diabetes, depression or dementia, decreased the odds of receiving an MCA; having hypertension increased the odds; and having chronic obstructive pulmonary disease/asthma had no clinically meaningful effect. In a US study, seniors who used a coupon for a free medication review were more likely to have high blood pressure (consistent with our finding), high cholesterol, or diabetes (inconsistent with our finding) [14]. Ontario residents with diabetes are eligible for MC Diabetes, and that is likely why having diabetes decreased the probability of getting an MCA. Nevertheless, despite the availability of MC Diabetes, a considerable number of individuals with diabetes received an MCA.

We assessed medication adherence, one of the intended outcomes of MCA [10], for three types of medications (ACEi, ARBs, statins) that three-fourths of our study population was receiving. We found that adherence with these drugs was high in the overall study population, irrespective of receiving an MCA. This potentially compromised our ability to determine whether nonadherence was a factor in patient selection. A contrasting but also unexpected finding of an analysis of Medicare beneficiaries showed that HF or diabetes patients who had better adherence were somewhat more likely to be enrolled into MTM services, and more likely to have a CMR in particular [29]. The researchers postulated that better adherence was an indicator of a ‘healthy user’ effect, which would also mean that these patients would be more likely to pursue a health-seeking behavior such as medication reviews [30].

A hospitalization or ED visit in the last year had little or no effect on the likelihood of receiving an MCA. A hospitalization or ED visit within 60 days of the eligible claim-date, in contrast, increased the odds of getting an MCA by 7 %. It is possible that such patients also received new prescriptions at discharge and visited a pharmacy to have them dispensed, which triggered the MCA.

### Pharmacy factors

In a UK study, pharmacy ownership type was the most significant determinant of MUR uptake, with chain pharmacies more likely to conduct MURs than independent pharmacies [21]. Because Ontario administrative databases do not capture pharmacy ownership data, pharmacy claims volume was the only pharmacy-level characteristic in our study. We identified that although the majority of MCAs was provided in high-volume pharmacies, the likelihood of receiving an MCA decreased with increase in pharmacy volume A likely explanation for this observation is that the relative increase on the pharmacist’s dispensing workload due to a MCA in a higher volume pharmacy is disproportionately high compared to the increase on a lower volume. In other words, pharmacies dispensing a high volume of prescriptions are less likely to have slack time that could be devoted to medication management services such as MedsCheck.

### Community factors

Residents of rural or non-major urban areas were found to have 27 % lower odds of receiving an MCA on an eligible claim-date compared to those living in major urban areas. Overall, 73 % of MCAs were delivered to patients who lived in urban areas as opposed to patients from non-major urban and rural areas. Similarly, in a US study, distance to the nearest pharmacy was negatively associated with the probability of getting a brown bag review where one mile increase in distance decreased the probability by 4 % [14]. An analysis of the Nova Scotia Seniors Pharmacare Medication Review program found that 75 % of pharmacies that provided the service were urban [31]. Consistent with findings in a UK study^22^ neighborhood income and material deprivation were negatively associated with receiving an MCA; however, the magnitude of the effect was not meaningful. Further studies with patient-level SES data would be needed to establish association with receiving MCA.

### Limitations

Although administrative data provide a powerful tool to answer research questions in a real-world setting they are also associated with a number of limitations. The fact that the ODB plan covers predominantly seniors has prevented us from evaluating the use of MCA in the entire population of MCA recipients. Also, in this study we had to deviate slightly on the eligibility definition compared to the MC program, as the original definition of the MC program required a senior to be on three medications for a chronic condition, while we assumed that the definition of a MC program required three chronic prescription medications. In addition, given that the ODB database captures only information about publicly-funded medications, prescription medications covered by private insurance could not be considered. The points above indicate that a small number of claim-dates could have been misclassified as ineligible. However, previous work from our group [11] has identified that most seniors who are <65 are on more than three chronic medications. Furthermore, pharmacists are aware that the MOHLTC does pharmacy audits for MCAs delivered to potentially ineligible individuals. Therefore, we expect that despite the variation on the definition of chronic medication and the inclusion of only publicly-funded medications, it is unlikely that an individual is receiving a MCA when not eligible. Finally, as described earlier, our database does not include any prescription claims covered by a private insurance plan. Should there have been more private pay seniors in the MCA group the estimated effect of the number and type of prescription drugs on receiving an MCA would be biased. However, there is currently no evidence that MCA recipients are more likely to be privately insured. However, there is currently no evidence that MCA recipients truly are more likely to be privately insured.

We identified and excluded a number of claims submitted after the date of death of an individual. In Ontario, it is legal for a healthcare provider to submit a claim for health services after the date of death of the individual. In addition, the proportion of such claims in our dataset was so small (<0.1 %) that we are confident that by excluding them we do not introduce any significant bias. We also implicitly assumed that on an eligible claim date patients or their caregivers^1^ had physically visited the pharmacy and were therefore “at risk” of receiving an MCA. However, that might not be the case for patients who chose to have their medication delivered at home or whose medications were picked up by a caregiver who did not have the patient’s consent to participate in an MCA. To minimize the effect of this assumption on study results, we excluded residents in long-term care facilities, and included dementia and depression (conditions typically limiting patients to their home) as covariates in the adjusted analysis. Nevertheless, there is still a probability that some of the eligible claim-dates refer to dates where no physical visit of the patient or eligible caregiver occurred. Future research should be directed at incorporating additional information that would help discerning whether a physical visit indeed would even be possible on an eligible claim-date. Examples of such information would include measures of frailty as well as the receipt of home care services. Similarly, it is impossible to know from the administrative databases whether the service was requested by the patient or was initiated by the pharmacist. Therefore, for those eligible patients who did not receive an MCA, it is unclear if it was not offered at all or if it was declined by the patient. We tried to control for the effect of patients’ willingness to receive a MCA by adjusting for medication adherence, a proxy for a ‘healthy user’ effect [30].

## Conclusion

In conclusion, the MCA program appears to have a mixed record of reaching those seniors most likely to benefit from a medication review. Seniors prescribed a new or high-risk medication or those who had been recently hospitalized were more likely to receive an MCA. In contrast, and at odds with our conceptual framework, older seniors and those with multiple and potentially inappropriate medications were less likely to receive an MCA. Whether this was due to the patient’s physical and/or mental inability to participate in a medication review in the pharmacy or to pharmacists’ avoidance of complicated patients will require additional study. If the barrier to access is related to patients’ characteristics, concerted efforts should be made by policy makers and practitioners to promote MCAs to caregivers and to recommend MedsCheck at Home for homebound patients. If the barrier is attributable to pharmacists directing their efforts towards less vulnerable eligible recipients, pharmacy and policy makers may wish to consider strategies to better align program eligibility criteria with patient need.

## Abbreviations

ADG: Aggregated Diagnosis Groups
CAN: Canadian
CMR: Comprehensive medication review
DIN: Drug identification numbers
ED: Emergency Department
GEE: Generalized Estimating Equations
HF: Heart failure
ICES: Institute for Clinical Evaluative Sciences
LTC: Long-term Care
MC: Medscheck
MCA: MedsCheck annual
MOHLTC: Ontario Ministry of health and Long-term Care
MTM: Medication therapy management
MUR: Medicines Use Reviews
ODB: Ontario Drug Benefit
OPEN: Ontario Pharmacy Research Collaboration
OR: Odds Ratio
QIC: Quasi-likelihood under the independence model criterion
RIO: Rurality Index of Ontario
SDS: Supplemental Digital Content
SES: Socio-economic status
UK: United Kingdom
US: United States
USA: United States of America

## Footnotes

1 Caregivers are eligible to receive an MCA with signed consent from the patient [10].
